# The Removal of Airborne Severe Acute Respiratory Syndrome Coronavirus 2 (SARS-CoV-2) and Other Microbial Bioaerosols by Air Filtration on Coronavirus Disease 2019 (COVID-19) Surge Units

**DOI:** 10.1093/cid/ciab933

**Published:** 2021-10-30

**Authors:** Andrew Conway Morris, Katherine Sharrocks, Rachel Bousfield, Leanne Kermack, Mailis Maes, Ellen Higginson, Sally Forrest, Joana Pereira-Dias, Claire Cormie, Tim Old, Sophie Brooks, Islam Hamed, Alicia Koenig, Andrew Turner, Paul White, R Andres Floto, Gordon Dougan, Effrossyni Gkrania-Klotsas, Theodore Gouliouris, Stephen Baker, Vilas Navapurkar

**Affiliations:** 1 The John Farman ICU, Cambridge University Hospitals, National Health Service (NHS) Foundation Trust, Cambridge, United Kingdom; 2 University Division of Anaesthesia, Department of Medicine, University of Cambridge School of Clinical Medicine, Cambridge Biomedical Campus, Cambridge, United Kingdom; 3 Department of Infectious Diseases, Cambridge University Hospitals NHS Foundation Trust, Cambridge, United Kingdom; 4 Clinical Microbiology Laboratory, Cambridge University Hospitals NHS Foundation Trust, Cambridge, United Kingdom; 5 Cambridge Institute of Therapeutic Immunology and Infectious Disease, Department of Medicine, University of Cambridge School of Clinical Medicine, Cambridge Biomedical Campus, Cambridge, United Kingdom; 6 Department of Clinical Engineering, Cambridge University Hospitals NHS Foundation Trust, Cambridge, United Kingdom; 7 Medical Technology Research Centre and School of Medicine, Anglia Ruskin University, Chelmsford, United Kingdom; 8 Molecular Immunity Unit, University of Cambridge Department of Medicine, MRC-Laboratory of Molecular Biology, Cambridge, United Kingdom; 9 Cambridge Centre for Lung Infection, Royal Papworth Hospital, Cambridge, United Kingdom

**Keywords:** SARS-CoV-2, air filtration, COVID-19, nosocomial infection, airborne pathogens

## Abstract

Airborne severe acute respiratory syndrome coronavirus 2 (SARS-CoV-2) was detected in a coronavirus disease 19 (COVID-19) ward before activation of HEPA-air filtration but not during filter operation; SARS-CoV-2 was again detected following filter deactivation. Airborne SARS-CoV-2 was infrequently detected in a COVID-19 intensive care unit. Bioaerosol was also effectively filtered.

Airborne dissemination is likely an important transmission route for severe acute respiratory syndrome coronavirus 2 (SARS-CoV-2) [1], with SARS-CoV-2 RNA detected in air samples from coronavirus disease 2019 (COVID-19) wards [1, 2]. Despite the use of personal protective equipment (PPE), there are multiple reports of patient-to-healthcare worker transmission of SARS-CoV-2 [3], potentially through the inhalation of viral particles [4]. There is a need to improve the safety for healthcare workers and patients by decreasing airborne transmission of SARS-CoV-2 [4]. Portable air filtration systems, which combine high efficiency particulate filtration and ultraviolet (UV) light sterilization, may be a scalable solution for removing respirable SARS-CoV-2 [5]. A recent review by the UK Scientific Advisory Group for Emergencies modeling group found limited data regarding the effectiveness of such devices [6]. Here we present the first data providing evidence for the removal of SARS-CoV-2 and microbial bioaerosols from the air using portable air filters with UV sterilization on a COVID-19 ward.

## METHODS

**Figure 1.**
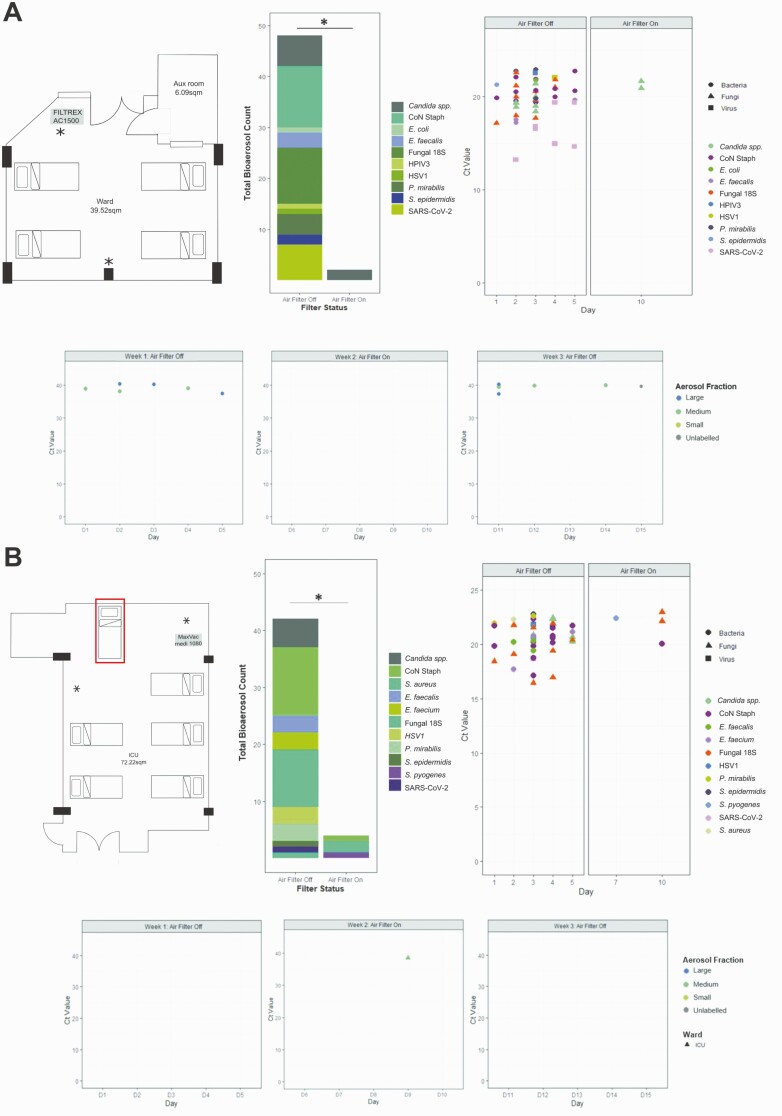
Bioaerosol detection in specific air sampler fractions over the 3-week testing period on a “surge” ward and “surge” ICU. *A*, Data from “surge” ward. Top left: Layout of the room on the “surge” ward with 4 beds. Air filter was installed in the marked location and set to operate at 1000 m^3^/hour with a room volume of approximately 107 m^3^. Top middle: Stacked bar chart showing collated total number of bioaerosol detections during weeks 1 (filter off) and 2 (filter on). ∗*P*=.05 by Mann-Whitney *U* test. Top right: CT values of detected pathogens by high-throughput qPCR when filter switch on and off. Bottom: CT values for the single qPCR SARS-CoV-2 detection when filter switch on and off. *B*, Data from “surge” ICU. Top left: Layout on the “surge” ICU with 6 beds including the addition of a further supra-capacity bed to increase occupancy (labeled with red box). Air filter was installed in the marked location and set to operate at 1000 m^3^/hour with a room volume of approximately 195 m^3^. Top middle: Stacked bar chart showing collated total number of bioaerosol detections during weeks 1 (filter off) and 2 (filter on) ∗*P*=.05 by Mann-Whitney *U* test. Top right: CT values of detected pathogens by high-throughput qPCR when filter switch on and off. Bottom: CT values for the single qPCR SARS-CoV-2 detection when filter switched on and off. NB: variation in CT values is a function of the microfluidics technology and do not reflect higher bioaerosol burdens. Abbreviations: CT, cycle threshold; ICU, intensive care unit; qPCR, quantitative polymerase chain reaction; SARS-CoV-2, severe acute respiratory syndrome coronavirus 2.

The study was conducted in 2 repurposed COVID-19 units in Addenbrooke’s Hospital, Cambridge, United Kingdom. One area was a “surge ward” (ward) managing patients requiring simple oxygen therapy or no respiratory support, the second was a “surge intensive care unit” (ICU) managing patients requiring invasive and noninvasive (noninvasive ventilation, high flow nasal oxygen) respiratory support. The ward was a fully occupied 4-bedded bay (Figure 1A top left panel). The ICU was fully occupied 5-bedded bay, with a supra-capacity sixth occupied bed used in week 2 (Figure 1B top left panel). Both units were passively ventilated, with 2–4 air-changes per hour at baseline.

In the ward we installed an AC1500 HEPA14/UV sterilizer (Filtrex, Harlow, UK); in the ICU we installed a Medi 10 HEPA13/UV sterilizer (Max Vac, Zurich, Switzerland). The air filters were placed in fixed positions before the initiation of the three-week study period (Figure 1A and 1B), switched on at the beginning of week 2 and run continuously from Sunday to Sunday for 24 hours per day, providing approximately 5–10 room-volume filtrations per hour.

We performed a crossover evaluation, with the primary outcome being detection of SARS-CoV-2 RNA in the various size fractions of air samples. Air sampling was conducted using National Institute for Occupational Safety and Health (NIOSH) BC 251 2-stage cyclone aerosol samplers [7] (B Lindsley, Centers for Disease Control and Prevention [CDC]), operated in accordance with previous studies [7, 8]. Air samplers were assembled daily with a control sampler left in a sealed bag. Samplers were placed adjacent to the air filter inlet and the other at approximately 4 meters from the filter and no closer than 2 meters to patients. In ICU 2 distant samplers were used: 1 mounted at head height and 1 at bed height. Samplers were operated on weekdays (from 08:15 to 14:15) for 3 consecutive weeks. After sampling, samplers were disassembled using sterile technique. The samples were processed and then stored at −80°C until analysis

Nucleic acids were extracted from each NIOSH sampler component (tubes containing large aerosols, medium aerosols, and filter). Methodological details including extractions, reverse transcription quantitative polymerase chain reaction (RT-qPCR) for SARS-CoV-2 and high throughput qPCR assays for a range of bacterial, viral, and fungal pathogens are in the Supplementary Methods (organisms listed in Supplementary Table 1). Differences in numbers of pathogens detected with filters on and off were compared by Mann-Whitney *U* test, and *P*≤.05 was considered significant.

## RESULTS

From 18 January to 5 February beds in the ward and ICU were at 100% occupancy; 15 patients were admitted to the ward, and 14 were admitted to the ICU over the sampling period. All patients were symptomatic and tested positive for SARS-CoV-2 RNA.

In the ward, during the first week while the air filter was inactive, we were able to detect SARS-CoV-2 on all sampling days; RNA was detected in both the medium (1–4 μM) and the large (>4 μM) particulate fractions (Figure 1A lower panel). SARS-CoV-2 RNA was not detected in the small (<1 μM) particulate filter. The air filter was run continuously in week 2; we were unable to detect SARS-CoV-2 RNA in any of the sampling fractions on any of the 5 testing days. We completed the study by repeating the sampling with an inactive air filter. As in week 1, we were able to detect SARS-CoV-2 RNA in the medium and the large particulate fractions on 3/5 days of sampling (a sample without tube size indicated tested positive on day 5) (Figure 1A lower panel). SARS-CoV-2 RNA was not detected from the control sampler.

We subjected the extracted nucleic acid preparations to high-throughput qPCR to detect a range of viral, bacterial, and fungal targets. In week 1, we detected nucleic acid from multiple viral, bacterial, and fungal pathogens on all sampling days (Figure 1A top, middle, and right panels). In contrast, when the air filter was switched on, we detected yeast targets only on a single day, with a significant reduction (*P*=.05) in microbial bioaerosols when the air filter was operational (Figure 1A). Using this high-throughput approach, SARS-CoV-2 RNA was detected on 4/5 days tested in week 1 but was again absent in week 2. We were unable to generate multiplex data for week 3 due to sample degradation following SARS-CoV-2 RNA amplification.

In contrast to the ward, we found limited evidence of airborne SARS-CoV-2 in ICU in weeks 1 and 3 (filter off) but detected SARS-CoV-2 RNA in a single sample in the medium (1–4 μM) particulates on week 2 (filter on) (Figure 1B lower panel). This contrary result did not reflect a general lack of bioaerosols in the ICU, which demonstrated a comparable quantity and array of pathogen associated nucleic acids to that seen in the unfiltered ward air on week 1 (Figure 1B top, middle, and right panels). Again, the use of the air filtration device significantly (*P*=.05) reduced the microbial bioaerosols (Figure 1B); with only 3 organism types detected on 2 of the sampling days. SARS-CoV-2 RNA was only detected once during week 1 on the high throughput qPCR assay.

## DISCUSSION

Our study represents the first report to our knowledge of removal of airborne SARS-CoV-2 in a hospital environment using combined air filtration and UV sterilization technology. Specifically, we provide evidence for the circulation of SARS-CoV-2 in a ward within airborne droplets of >1 μM. Droplets of 1–4 μM are likely a key vehicle for SARS-CoV-2 transmission [9], as they remain airborne for a prolonged period and can deposit in the distal airways. Recent data have shown that exertional respiratory activity, such as that seen in patients with COVID-19, increases the release of 1–4 μM respiratory aerosols, relative to conventionally defined “aerosol generating procedures” such as noninvasive respiratory support [10]. Patients in ICU are commonly at a later stage of disease and may shed less virus as a result. These data are consistent with our observations, suggesting that aerosol precautions may be more important in conventional wards than in well-defined “aerosol risk areas.”

The sampling and detection of airborne viruses poses several technological challenges, and there remains no agreed standard for their use or interpretation [11]. However, the detection of SARS-CoV-2 RNA by RT-qPCR (albeit at a high cycle threshold [CT] value), and the lack of detection during use of an air sterilization system, adds to a growing body of evidence implicating the airborne transmission of SARS-CoV-2 [1]. The detection of SARS-CoV-2 RNA in the air of a ward managing patients with COVID-19 intimates that this is a key mechanism by which healthcare professionals could become infected. The removal of airborne viral particles and other pathogens may help reduce the likelihood of hospital-acquired respiratory infections. This reduction may be by both decreasing the load of respirable particles and by removal of larger droplets that can facilitate fomite-associated spread [11]. The clearance of bioaerosol was not restricted to SARS-CoV-2. Although the impact of air filtration on nosocomial infection is uncertain [5, 12], the broad range of pathogens removed in this study suggests potential for benefit beyond SARS-CoV-2.

This study has limitations. The evaluation was conducted in 2 rooms, and there are no data defining the optimal air changes required to remove detectable pathogens with the specified devices, nor their impact in better ventilated facilities. Given the large volume of air within the room and the stability of viruses in the sampling fluid, it was predictable that the amount of SARS-CoV-2 detected would be minimal. However, negative results from the control sampler—and the striking but reversible effect of the air filtration devices—suggest these are not false positive detections, and we cannot exclude the risk of airborne infection. Future studies should examine whether air filtration devices have an impact on healthcare professional and patient focused outcomes, including measuring infection/exposure as an endpoint, as well as assessing potential harm, such as noise, reduced ambient humidity, or impact on delivery of care.

We were able to detect airborne SARS-CoV-2 RNA in a repurposed COVID-19 “surge ward” and found that air filtration can remove SARS-CoV-2 RNA below the limit of qPCR detection. SARS-CoV-2 was infrequently detected in the air of a “surge ICU”; however, the device retained its ability to reduce microbial bioaerosols. Portable air filtration devices may mitigate the reduced availability of airborne infection isolation facilities when surges of COVID-19 patients overwhelm healthcare resources and improve safety of those at risk of exposure to respiratory pathogens such as SARS-CoV-2.

## Supplementary Data

Supplementary materials are available at *Clinical Infectious Diseases* online. Consisting of data provided by the authors to benefit the reader, the posted materials are not copyedited and are the sole responsibility of the authors, so questions or comments should be addressed to the corresponding author.

ciab933_suppl_Supplementary_Material

ciab933_suppl_Supplementary_Data_S1

ciab933_suppl_Supplementary_Data_S2

## Notes

***Author contributions.***

A. C. M.: Conceptualization, methodology, data analysis, and writing original draft.

K. S.: Investigation, supervision, writing review, and editing.

R. B.: Investigation, supervision, writing review, and editing.

L. K.: Investigation, data analysis, supervision, writing review, and editing.

M. M.: Investigation, writing review, and editing.

E. H.: Investigation, data analysis, writing review, and editing.

S. F.: Investigation, writing review, and editing.

J. D.: Investigation, writing review, and editing.

T. O.: Investigation, writing review, and editing.

S. Br.: Investigation, writing review, and editing.

I. H.: Investigation, writing review, and editing.

A. K.: Investigation, writing review, and editing.

A. T.: Investigation, writing review, and editing.

P. W.: Conceptualization, provision of resources, writing review, and editing.

A. F.: Provision of resources, writing review, and editing.

G. D.: Conceptualization, provision of resources, supervision, writing review, and editing.

E. G.: Conceptualization, supervision, investigation, writing review, and editing.

T. G.: Conceptualization, supervision, investigation, writing review, and editing.

S. B.: Conceptualization, provision of resources, supervision, data analysis, and writing original draft.

V. N.: Conceptualization, provision of resources, supervision, data analysis, and writing original draft.

Data for organisms detected by single plex PCR (SARS-CoV-2) and high throughput PCR (bioaerosol) are included as supplementary data files.

***Financial sources.*** This work was supported by a Wellcome Trust senior research fellowship to Stephen Baker (grant number 215515/Z/19/Z), and National Institute for Health Research Antimicrobial Resistance (NIHR AMR) Research Capital Funding Scheme (grant number NIHR 200640). A. C. M. is supported by a Clinician Scientist Fellowship from the Medical Research Council (grant number MR/V006118/1). M. M. and S. F. are funded by the National Institute for Health Research (Cambridge Biomedical Research Centre at the Cambridge University Hospitals NHS Foundation Trust). The views expressed are those of the authors and not necessarily those of the NHS, the NIHR, or the Department of Health and Social Care. The funders had no role in the design and conduct of the study; collection, management, analysis, and interpretation of the data; preparation, review, or approval of the manuscript; and decision to submit the manuscript for publication. The study was registered as a service evaluation with Cambridge University Hospitals NHS Foundation Trust (service evaluation number PRN 9798).

***Potential conflicts of interest.*** V. N. is the founder, director, and shareholder of Cambridge Infection Diagnostics Ltd. A. C. M., P. W., G. D., and S. B. are members of the Scientific Advisory Board of Cambridge Infection Diagnostics Ltd. T. G. has received a research grant from Shionogi in materials for antimicrobial susceptibility testing in 2021 unrelated to this study and collaborated with Inivos in a PPE decontamination project (materials provided by the company unrelated to this study). R. A. F. has received research grants and/or consultancy payments from UK Cystic Fibrosis Trust during and outside of the submitted work, Wellcome Trust, Medical Research Council, Foundation Botnar, GlaxoSmithKline, AstraZeneca, Chiesi, Shionogi, Insmed, Thirty Technology, and received NIOSH air samplers provided by B. Lindsey (US CDC). E. G.-K. has received a National Institute of Health Research Greenshoots Award and is a NIHR Clinical Research Network Lead; fees for ECCMID meeting registration from ESCMID; unpaid roles for the European Society of Clinical Infectious Diseases and Microbiology COVID 19 guidelines writing group member and guidelines committee member. G. D. received financial or nonfinancial interests from Microbiotica Ltd and had a leadership or fiduciary role in other board, society, committee, or advocacy group, paid or unpaid for Wellcome. All other authors report no potential conflicts. All authors have submitted the ICMJE Form for Disclosure of Potential Conflicts of Interest. Conflicts that the editors consider relevant to the content of the manuscript have been disclosed. This work is licensed under a Creative Commons Attribution 4.0 International License.
